# Optimizing a Classification Model to Evaluate Individual Susceptibility in Noise-Induced Hearing Loss: Cross-Sectional Study

**DOI:** 10.2196/60373

**Published:** 2024-11-14

**Authors:** Shiyuan Li, Xiao Yu, Xinrong Ma, Ying Wang, Junjie Guo, Jiping Wang, Wenxin Shen, Hongyu Dong, Richard Salvi, Hui Wang, Shankai Yin

**Affiliations:** 1 Department of Otolaryngology–Head and Neck Surgery, Shanghai Sixth People’s Hospital, Shanghai Jiao Tong University School of Medicine, 600 Yishan Road, Shanghai, 200030, China, 86 18060587551; 2 Otolaryngology Institute, Shanghai Jiao Tong University, Shanghai, China; 3 Shanghai Key Laboratory of Sleep Disordered Breathing, Shanghai, China; 4 Center for Hearing and Deafness, University at Buffalo, Buffalo, NY, United States

**Keywords:** noise-induced hearing loss, susceptible, resistance, machine learning algorithms, linear regression, extended high frequencies, phenotypic characteristics, genetic heterogeneity

## Abstract

**Background:**

Noise-induced hearing loss (NIHL), one of the leading causes of hearing loss in young adults, is a major health care problem that has negative social and economic consequences. It is commonly recognized that individual susceptibility largely varies among individuals who are exposed to similar noise. An objective method is, therefore, needed to identify those who are extremely sensitive to noise-exposed jobs to prevent them from developing severe NIHL.

**Objective:**

This study aims to determine an optimal model for detecting individuals susceptible or resistant to NIHL and further explore phenotypic traits uniquely associated with their susceptibility profiles.

**Methods:**

Cross-sectional data on hearing loss caused by occupational noise were collected from 2015 to 2021 at shipyards in Shanghai, China. Six methods were summarized from the literature review and applied to evaluate their classification performance for susceptibility and resistance of participants to NIHL. A machine learning (ML)–based diagnostic model using frequencies from 0.25 to 12 kHz was developed to determine the most reliable frequencies, considering accuracy and area under the curve. An optimal method with the most reliable frequencies was then constructed to detect individuals who were susceptible versus resistant to NIHL. Phenotypic characteristics such as age, exposure time, cumulative noise exposure, and hearing thresholds (HTs) were explored to identify these groups.

**Results:**

A total of 6276 participants (median age 41, IQR 33‐47 years; n=5372, 85.6% men) were included in the analysis. The ML-based NIHL diagnostic model with misclassified subjects showed the best performance for identifying workers in the NIHL-susceptible group (NIHL-SG) and NIHL-resistant group (NIHL-RG). The mean HTs at 4 and 12.5 kHz showed the highest predictive value for detecting those in the NIHL-SG and NIHL-RG (accuracy=0.78 and area under the curve=0.81). Individuals in the NIHL-SG selected by the optimized model were younger than those in the NIHL-RG (median 28, IQR 25‐31 years vs median 35, IQR 32‐39 years; *P*<.001), with a shorter duration of noise exposure (median 5, IQR 2‐8 years vs median 8, IQR 4‐12 years; *P*<.001) and lower cumulative noise exposure (median 90, IQR 86‐92 dBA-years vs median 92.2, IQR 89.2‐94.7 dBA-years; *P*<.001) but greater HTs (4 and 12.5 kHz; median 58.8, IQR 53.8‐63.8 dB HL vs median 8.8, IQR 7.5‐11.3 dB HL; *P*<.001).

**Conclusions:**

An ML-based NIHL diagnostic model with misclassified subjects using the mean HTs of 4 and 12.5 kHz was the most reliable method for identifying individuals susceptible or resistant to NIHL. However, further studies are needed to determine the genetic factors that govern NIHL susceptibility.

## Introduction

Noise-induced hearing loss (NIHL), one of the leading occupational health concerns worldwide, is second only to presbycusis as a cause of hearing loss [1]. Approximately 1.3 billion people experience hearing loss due to noise exposure [2]. The problem has been exacerbated by a rise in recreational noise exposure through prolonged use of headphones and attendance at loud music venues, especially among younger populations [3]. In order to strengthen prevention and mitigate its escalating public health impact, it is important to identify people who are very susceptible to noise damage.

Although NIHL is generally considered an acquired type of hearing loss, a multifactorial interaction between intrinsic (genetic) and external (environmental) factors is likely involved in the development of NIHL [4,5]. The genetic contribution is supported by the fact that individuals exposed to the same noise show great variation in their susceptibility [6]. Some people developed a large amount of elevation in hearing thresholds (HTs) only after a short period of noise exposure at lower levels. They are considered susceptible individuals, in contrast to their noise-resistant counterparts who get little or no elevation in HTs after a long period of noise exposure at high levels [7]. Classifying people who are susceptible to NIHL (referred to as the NIHL-susceptible group [NIHL-SG]) from those who are resistant (referred to as the NIHL-resistant group [NIHL-RG]) is important for both research and noise safety management. Genetic comparison between the 2 groups could help to identify genes associated with NIHL susceptibility [8]. In preventative noise exposure management, it is obviously beneficial to minimize future hearing loss by removing the people in the NIHL-SG from jobs with heavy noise exposure.

The classification of people susceptible to NIHL has been the aim of many studies. However, there was no clear scientific consensus for such classification to pinpoint susceptibility. Some have classified the NIHL-SG as those with HTs in the upper 10% or 20% at 3, 4, or 6 kHz; those with HTs in the lower 10% or 20% defined the NIHL-RG [9-11]. Others have used linear regression and quadratic regression models, considering the HTs residual values as classification indicators [12,13]. Interestingly, machine learning (ML) has been used to predict audiometric classifications, with misclassified subjects grouped into the NIHL-SG or NIHL-RG [8]. One limitation of prior studies is that they primarily focused on the degree of HT elevation, neglecting other risk factors such as age, gender, health, and noise exposure intensity. Although people who were identified with a high risk of NIHL in those studies were all those who had large amounts of NIHL, they were also those who had larger cumulative noise exposure (CNE). Other limitations in the field of NIHL include small sample sizes or inadequate population representation. Thus, the optimal method for reliably identifying the NIHL-SG and NIHL-RG remains a significant challenge.

NIHL typically shows a “notched” audiogram with the worst thresholds around 3‐6 kHz. Most prior studies attempting to distinguish susceptible versus resistant individuals have relied solely on this frequency region [9-11]. However, recent evidence suggests that monitoring hearing loss at extended high frequencies (EHFs) from 9‐20 kHz may reveal noise-related impairment earlier or deteriorate more rapidly than at conventional clinical test frequencies [14-17]. Few studies have leveraged EHFs to classify NIHL susceptibility. ML techniques enable the modeling of nonlinear relationships between multiple factors and HTs measured across a wide range of frequencies, making it possible to identify the most reliable frequencies [18]. Identifying the most reliable frequencies through ML could enhance the early detection accuracy of those vulnerable to occupational noise exposures.

Thus, this study aimed to determine an optimal model and reliable audiometric frequencies for identifying the NIHL-SG and NIHL-RG. Afterward, the clinical profiles of these 2 groups were compared to identify risk factors.

## Methods

### Study Setting

This cross-sectional study was conducted from June 2015 to June 2021 at 2 shipyards in Shanghai, China. The study recruited employees who underwent annual occupational health examinations due to exposure to high noise levels from activities such as sanding, welding, metalworking, and cutting. Over 99% of the recruited employees were from Eastern China and predominantly of Han ethnicity. During the annual examinations, the employees completed a detailed questionnaire that collected demographic information, noise exposure history, job type, smoking and alcohol habits, history of serious illnesses (including hereditary and drug-related hearing loss), use of hearing protection devices, and exposure to other harmful chemicals.

### Data Collection and Study Procedure

Of the 9669 participants who completed the questionnaire, 6276 individuals were included in the final analysis after applying the exclusion criteria of preexisting hearing loss (≥25 dB HL at 0.5‐6 kHz) on their preemployment audiogram, career length with industrial noise exposure <1 year, otologic diseases, history of hereditary deafness and abnormal middle ear impedance, toxic substances exposure, and other invalid information.

**Figure 1.**
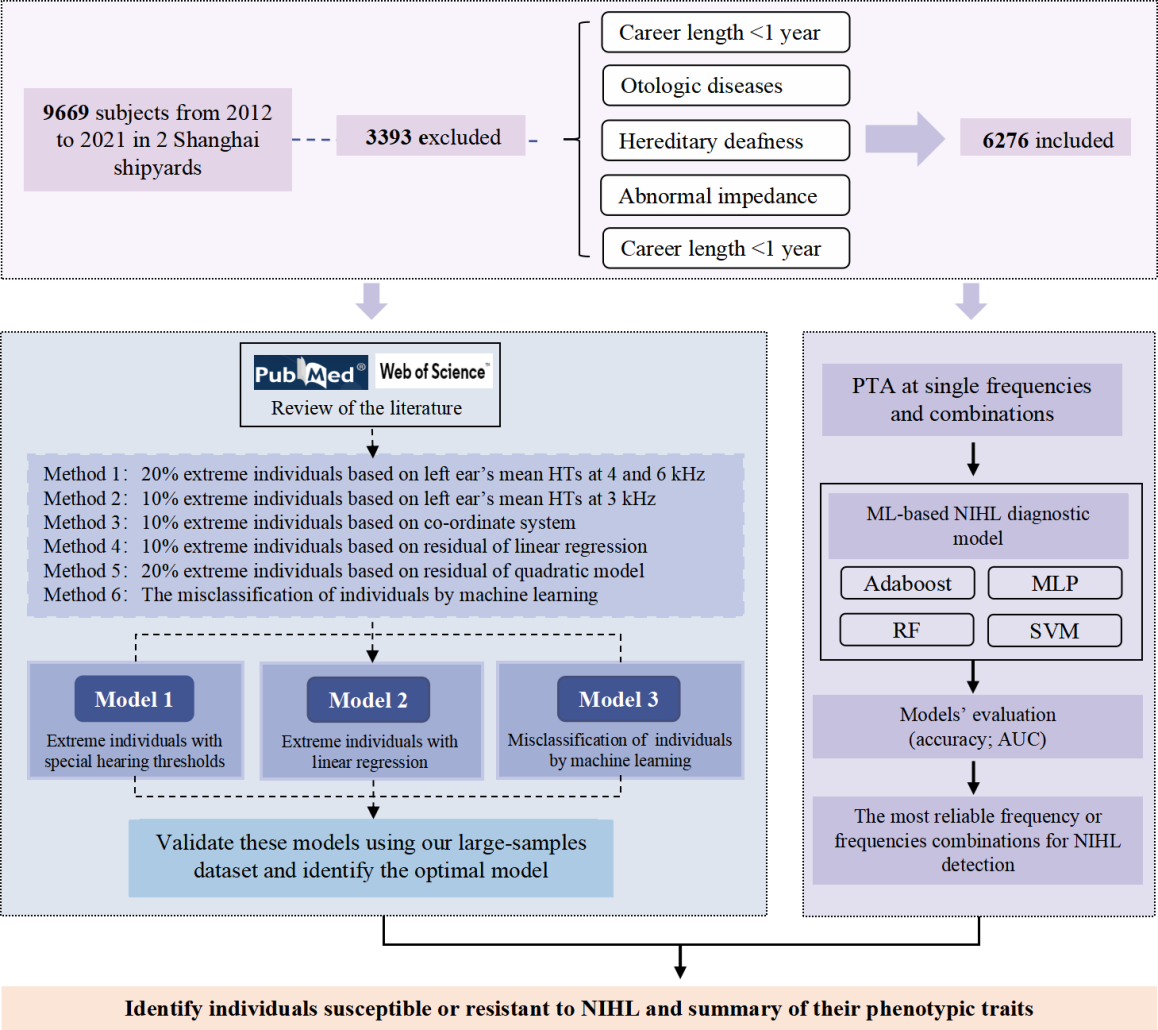
Flowchart of the study. Adaboost: adaptive boosting; AUC: area under the curve; HTs: hearing thresholds; ML: machine learning; MLP: multilayer perceptron; NIHL: noise-induced hearing loss; PTA: pure tone audiometry; RF: random forest; SVM: support vector machine.

Figure 1 briefly outlines the study procedure. We first summarize previously published methods for classifying the NIHL-SG and NIHL-RG. Then, each method was applied to our dataset to assess classification performance. The optimal classification method was identified by comparing the classified group assignments to known characteristics associated with NIHL, that is, a form of face validity where the assigned category measures what it purports to measure. An ML diagnostic model was constructed to determine the predictive value of various frequencies or combinations of audiometric frequencies. Finally, the optimal method, with the participants of this research and the most reliable frequencies, was used to form the NIHL-SG and NIHL-RG. Demographic and clinical features of each group were analyzed to identify traits uniquely associated with susceptibility profiles.

### Ethical Considerations

This study was reviewed and approved by the institutional ethics review board at Shanghai Sixth People’s Hospital affiliated with Shanghai Jiao Tong University (2017‐136) and was registered in the Chinese Clinical Trial Registry under the identifier ChiCTR-RPC-17012580. The aims and procedures of the study were explained, and written informed consent was obtained from each participant prior to the study. The original informed consent allows for secondary analysis without additional consent. The study data were deidentified to safeguard the privacy of the participants. Compensation was not provided for participants in this research.

### Audiological Evaluation and Noise Exposure Estimation

The hearing evaluation was performed by a qualified medical assistant. An otoscopy inspection was carried out to rule out blockage of the external ear. Tympanometry was tested with a TympStar tympanometer (Grason-Stadler) to ensure normal middle ear impedance, which was indicated by a type A tympanogram (peak between −100 and +100 daPa).

Industrial noise levels were assessed using an ASV5910-R digital recorder (Aihua Instruments) across different work areas, adhering to the national standard of China [19]. Evaluation of noise exposure used the CNE formula:

 CNE=Leq-8h+10*log(T)

Here, Leq-8h signifies the equivalent continuous sound level for 8 hours and *T* denotes the exposure time [20]. Detailed information is presented in Multimedia Appendix 1.

### Review of Published Methods

An initial literature search was conducted on PubMed and Web of Science, yielding 597 records related to NIHL. After a comprehensive examination of the details, 6 eligible studies were finally grouped into 1 of 3 models based on the approach. Detailed information is presented in Multimedia Appendix 1.

### Determining the Optimal Method to Create the NIHL-SG and NIHL-RG

The 6 methods of the 3 models were independently applied to the study data according to the original inclusion and exclusion criteria, subgroup definitions, and frequency thresholds specified. This replication of all prior published methods ensured a valid like-for-like assessment. The predicted NIHL-SG and NIHL-RG from each model were then characterized in terms of demographics, noise exposure histories, and audiometric profiles. Based on general trends, susceptible individuals were generally younger, had lower CNE, shorter exposure duration, and elevated HTs compared to their resistant counterparts. The agreement between each model’s classifications and these known NIHL-susceptible traits was analyzed. Consistency with characteristic risk factors like significantly younger age and higher noise doses in the predicted SG indicated accurate classification (ie, good face validity) of risk. Conversely, inconsistencies with these known risk factors in the predicted groups were interpreted as poorer predictive validity (ie, poor face validity). Using statistical tests of differences, the model exhibiting the strongest concordance with established susceptible characteristics was deemed the optimal approach.

### Determining a Reliable Test Frequency for NIHL

A total of 4 ML algorithms (adaptive boosting, multilayer perceptron, random forest, and support vector machine) [21-24] were applied using HTs at different frequencies (4 and 12.5 kHz) and combinations of frequencies (4 with 12.5 kHz; 4, 6, and 12.5 kHz; 4, 10, and 12.5 kHz; 4, 6, 10, and 12.5 kHz; and 3, 4, 6, 10, and 12.5 kHz) as predictive inputs to develop an NIHL diagnostic model. A total of 5 demographic and lifestyle variables known to be significant for the risk of NIHL were also included as inputs to the ML programs—age [7], gender [25], CNE [26], smoking status [27], and alcohol consumption [28]. All models constructed by each algorithm were validated using 10-fold cross-validation. In each cross-validation iteration, 9 subsets were used as training data while the remaining 1 subset served as test data. This process was repeated 10 times to calculate the average accuracy and area under the curve (AUC) of each model. The single frequency and frequency combinations that exhibited the highest testing accuracy and AUC were determined to be the most reliable of NIHL susceptibility.

### Clinical Characteristics of the NIHL-SG and NIHL-RG

We first constructed an optimized model using the most reliable frequencies and methods. This model was then applied to the full dataset to predict the identification of individuals in the NIHL-SG or NIHL-RG. The characteristics of the individuals in the predicted NIHL-SG and NIHL-RG were then compared. Parameters evaluated included demographics (gender ratio and age), noise exposure histories (duration and intensity of noise exposure), lifestyle habits (proportions reporting smoking and alcohol use), and PTA for various frequencies. Linear regression lines were fitted between hearing loss and exposure time, with the intercept forced to 0.

### Statistical Analysis

Continuous data with a skewed distribution were reported as median and IQR values and analyzed using the Mann-Whitney test between groups. Categorical data were presented as counts and percentages and were compared using the Pearson chi-square test. ML algorithms were implemented using R software (version 4.02; R Core Team). Statistical analyses were conducted using SPSS 27.0 software (IBM) and Prism 8.3 (GraphPad Software Inc). Statistical significance was defined as *P*<.05.

## Results

### Demographics and Hearing Characteristics of Participants

The basic characteristics of the 6276 participants are shown in Table 1. The median (IQR) age was 41 (33‐47) years and the median (IQR) of CNE was 93.2 (89.2‐97.7) dBA-years. There were 2382 (38%) participants who currently smoked and 2275 (36.2%) who currently drank alcohol. The median (IQR) of PTAs was 16.7 (12.5‐21.7) dB at 0.5‐2 kHz, 22.5 (15‐40) dB at 3 kHz, 30 (17.5‐50) dB at 4 kHz, 25 (15‐42.5) dB at 6 kHz, 30 (17.5‐50) dB at 10 kHz, 47.5 (27.5‐67.5) dB at 12.5 kHz, and 40 (25‐55) dB at 4 and 12.5 kHz combined.

**Table 1.** Demographic characteristics and audiometric data of the participants (N=6276).

Variables	Participants
**Sex, n (%)**	
Male	5372 (85.6)
Female	904 (14.4)
Age (years), median (IQR)	41 (33‐47)
Exposure time (years), median (IQR)	7 (4‐11)
CNE^a^ (dBA-year), median (IQR)	93.2 (89.2‐97.7)
**Smoking, n (%)**	
Currently	2382 (38)
Never	3894 (62)
**Drinking, n (%)**	
Currently	2275 (36.2)
Never	4001 (63.8)
**PTA^b^ (dB HL, kHz), median (IQR)**	
0.5‐2	16.7 (12.5‐21.7)
3	22.5 (15.0‐40.0)
4	30.0 (17.5‐50.0)
6	25.0 (15.0‐42.5)
10	30.0 (17.5‐50.0)
12.5	47.5 (27.5‐67.5)
4 and 6	28.8 (17.5‐45.0)
4 and 12.5	40.0 (25.0‐55.0)
4, 6, and 12.5	35.0 (22.5‐49.2)
4, 10, and 12.5	36.7 (23.3‐52.5)
4, 6, 10, and 12.5	33.8 (21.9‐48.8)
3, 4, 6, 10, and 12.5	32.0 (21.0‐46.5)

a CNE: cumulative noise exposure.

b PTA: pure tone audiometry.

### Validation of Previous Screening Models

Detailed information is presented in Multimedia Appendix 1.

### Validation of Model 1 With Extreme Thresholds at 3, 4, and 6 kHz

In method 1, a total of 3276 individuals met the inclusion and exclusion criteria. The mean age of the NIHL-SG (n=655) was about 7 years older than the NIHL-RG (n=655; Figure S1 in Multimedia Appendix 1, Table 2; median 38, IQR 32-44 years vs median 31, IQR 27‐36.8 years; *P*<.001). PTA values at 4 and 6 kHz were significantly greater in the NIHL-SG than the NIHL-RG (Figure S1 in Multimedia Appendix 1, Table 2; median 55, IQR 47.5‐62.5 dB vs median 10, IQR 7.5‐12.5 dB; *P*<.001).

**Table 2.** Information on published methods for classifying people susceptible or resistant to noise-induced hearing loss (NIHL), along with the validation results of each method using our dataset.

Participants		HTs^a^ adjusted by other factors	Screening guideline	Validation results of our dataset				
				Group	Age (years), median (IQR)	Noise exposure duration (years), median (IQR)	CNE^b^ (dBA-years), median (IQR)	PTA^c^ (dB), median (IQR)
**Method 1** [9]								
	Exclusion criteria are (1) history of meningitis, (2) aminoglycoside treatment, (3) acoustic trauma, (4) the hearing gap between the right and the left ear <40 dB, (5) previous noise exposure <5 years, (6) female, (7) the duration of exposure to noise >([age × 0.666]–20 years)	Workers were divided into 9 categories: years of exposure ranges: <15, 15‐25, and >25 years; noise exposure levels: ≤85, 86-91, and ≥92 dBA	Selected the 20% most susceptible and the 20% most resistant participants based on the mean HTs for the left ear at 4 and 6 kHz from each category	NIHL-SG^d^ NIHL-RG^e^	38 (32‐44) 31 (27‐36.8)	10 (7‐14) 7 (4‐12)	93.4 (91‐96.2) 91.4 (87.4‐94.4)	55 (47.5‐62.5) at 4 and 6 kHz 10 (7.5‐12.5) at 4 and 6 kHz
**Method 2** [10]								
	Exclusion criteria are (1) history of head injury, otological disease, and other diseases that could affect hearing; (2) treatment with ototoxic drugs; (3) potentially harmful noise exposure; and (4) not using hearing protectors	Workers were divided into 9 categories: age-ranges:<35, 35‐50, and >50 years; noise exposure categories:<85, 86‐91, and >92 dBA	Selected the 10% most susceptible and the 10% most resistant participants based on the left ear’s mean HTs at 3 kHz from each category	NIHL-SG NIHL-RG	48 (41.25‐51.8) 42 (36‐51)	16 (15‐20) 16 (15‐18)	96.3 (94‐104.5) 96.2 (93.2‐98.2)	65 (60‐70) at 3 kHz 10 (5‐10) at 3 kHz
**Method 3** [11]								
	Exclusion criteria are (1) noise exposure <1 year, (2) history of middle ear diseases, (3) hearing impairment, (4) air-bone gap in pure-tone audiometry, (5) the difference between HTs in the right and the left ear at 4 and 6 kHz <40 dB	N/A^f^	Standardized HTs for the left ear of all participants were plotted on the Cartesian coordinate system (axis X – HT at 4 kHz and axis Y – HT at 6 kHz) and 10% of participants (points) were cut off at each extreme as the most susceptible or resistant individuals	NIHL-SG NIHL-RG	47 (40‐52) 31 (26‐36)	9 (6‐12) 5 (3‐10)	95.9 (92.2‐103.7) 91 (87.4‐93.4)	70 (65‐75) at 4 and 6 kHz 7.5 (5‐10) at 4 and 6 kHz
**Method 4** [13]								
	Inclusion criteria are (1) CNE >80 dBA and (2) work experience at noisy environment	N/A	Used simple linear regression of bilateral HTs at 3, 4, and 6 kHz versus CNE, assigning those with the 10% worst residuals and 10% best residuals (measured minus predicted) to the most resistant and susceptible individuals	NIHL-SG NIHL-RG	46 (40‐50) 35 (28.5‐41.5)	8 (5‐12) 7 (4‐11)	93.2 (90.2‐98) 95.9 (92.5‐100.7)	64.2 (58.3‐70) at 3, 4, and 6 kHz 18.8 (11.3‐27.5) at 3, 4, and 6 kHz
**Method 5** [12]								
	Inclusion criteria are (1) work experience at the mill under a noisy environment >1 year; (2) no work experience in other factories with noise; (3) no history of drug-related, genetic, or age-related hearing impairments, head wounds, or other ear diseases	N/A	Established a quadratic model between the CNE and estimated HTs at 3, 4, and 6 kHz. Classified 20% in the lowest quintile of residuals as NIHL-resistant, and individuals in the highest quintile of residuals as NIHL-susceptible	NIHL-SG NIHL-RG	49 (44‐53) 40 (33‐48.1)	9 (6‐13) 9 (5‐13)	101.1 (96.6‐103.7) 100.7 (97.7‐104.2)	61.7 (55.8‐67.5) at 3, 4, and 6 kHz 15 (11.7‐18.3) at 3, 4, and 6 kHz
**Method 6** [8]								
	Exclusion criteria are (1) career length <1 year, (2) otologic diseases or family history of deafness, (3) abnormal impedance, (4) toxic substances exposure, and (5) invalid information	Adjustments at the algorithmic level	Used age, sex, CNE, smoking, and alcohol drinking status as input variables, to predict hearing outcomes. The selection for the extreme individuals was restricted to the misclassified subjects by all algorithms. The NIHL-SG and NIHL-RG were comprised of 150 individuals with the largest probability values in each misclassified category provided by ML^g^ algorithms.	NIHL-SG NIHL-RG	32 (30‐34) 48 (45‐51)	6 (3‐10) 9 (5‐12)	91.5 (88.3‐94.7) 96.2 (92.2‐101.7)	33.8 (29‐43) at 3, 4, 6, 10, and 12.5 kHz 21 (16.9‐23.6) at 3, 4, 6, 10, and 12.5 kHz

a HT: hearing threshold.

b CNE: cumulative noise exposure.

c PTA: pure tone audiometry.

d NIHL-SG: noise-induced hearing loss susceptible group.

e NIHL-RG: noise-induced hearing loss resistant group.

f N/A: not applicable.

g ML: machine learning.

For method 2 analysis, only 681 individuals were included. The individuals classified as NIHL-SG on average were about 6 years older than those in NIHL-RG (Figure S1 in Multimedia Appendix 1, Table 2; median 48, IQR 41.25‐51.8 years vs median 42, IQR 36‐51 years; *P*=.006). They also showed a greater PTA at 3 kHz (Figure S1 in Multimedia Appendix 1, Table 2; median 65, IQR 60‐70 dB vs median 10, IQR 5‐10 dB; *P*<.001).

For method 3, a total of 5290 individuals were included in the analysis. The NIHL-SG (n=529) was significantly older (almost 20 years on average; Figure S1 in Multimedia Appendix 1, Table 2; median 47, IQR 40‐52 years vs median 31, IQR 26‐36 years; *P*<.001) and had notably poorer hearing than the NIHL-RG (n=529; Figure S1 in Multimedia Appendix 1, Table 2; median 70, IQR 65‐75 dB vs median 7.5, IQR 5‐10 dB; *P*<.001).

All individuals in the NIHL-SG selected by model 1 were older, had longer exposure time, and had higher CNE than those in NIHL-RG which is inconsistent with known characteristics (ie, poor validity). Several issues related to the analysis are of concern. There is no uniform criterion to define the age range or noise intensity range for different categories. Additionally, it is unclear how many or what percent of individuals should be selected to form the NIHL-SG and NIHL-RG. The ability of model 1 to identify individuals in the NIHL-SG and NIHL-RG is limited due to the potential influence of manual sorting.

### Validation of Model 2—Linear Regression CNE Versus Mean HTs

The 5460 individuals with CNE levels above 80 dBA were included in the analysis of method 4. The bilateral HTs at 3, 4, and 6 kHz versus CNE were significantly correlated (*R*^2^=0.102; *P*<.001). The NIHL-SG had slightly longer exposure times (Figure S2 in Multimedia Appendix 1, Table 2; median 8, IQR 5-12 years vs median 7, IQR 4‐11 years; *P*=.01) and higher HTs at 3, 4, and 6 kHz (Figure S2 in Multimedia Appendix 1, Table 2; median 64.2, IQR 58.3‐70 dB vs median 18.8, IQR 11.3‐27.5 dB; *P*<.001) compared to the NIHL-RG.

For method 5, a total of 2315 individuals met the inclusion and exclusion criteria. Quadratic regression of bilateral HTs at 3, 4, and 6 kHz versus CNE was significantly correlated (*r*^2^=0.078; *P*<.001). The NIHL-SG demonstrated a greater average hearing loss of nearly 47 dB at 3, 4, and 6 kHz compared to the NIHL-RG (Figure S2 in Multimedia Appendix 1, Table 2; median 61.7, IQR 55.8-67.5 dB vs median 15, IQR 11.7‐18.3 dB; *P*<.001); however, this may be related to the fact that the individuals in the NIHL-SG were significantly older than the NIHL-RG (Figure S2 in Multimedia Appendix 1, Table 2; median 49, IQR 44‐53 years vs median 40, IQR 33‐48.1 years; *P*<.001).

Although model 2 showed that HTs were correlated with CNE, lower *R*^2^ values indicate that these models have not precisely identified individuals highly susceptible or resistant to NIHL.

### Validation of Model 3 With ML

The ML model achieved an average accuracy of 0.74 and an average AUC of 0.80. Workers in the NIHL-SG (n=150) were younger than those in the NIHL-RG (n=150; Figure S3 in Multimedia Appendix 1, Table 2; median 32, IQR 30‐34 years vs median 48, IQR 45‐51 years; *P*<.001); received lower noise exposure levels (Figure S3 in Multimedia Appendix 1, Table 2; median 91.5, IQR 88.3-94.7 dBA-years vs median 96.2, IQR 92.2‐101.7 dBA-years; *P*<.001); had shorter noise durations (Figure S3 in Multimedia Appendix 1, Table 2; median 6, IQR 3‐10 years vs median 9, IQR 5‐12 years; *P*<.001) but greater HTs (Figure S3 in Multimedia Appendix 1, Table 2; median 33.8, IQR 29‐43 dB vs median 21, IQR 16.9‐23.6 dB; *P*<.001) than those in the NIHL-RG.

The misclassified subjects selected by the ML diagnostic model for the NIHL-SG and NIHL-RG were consistent with their expected clinical characteristics (ie, strong face validity). The ML model also maintained a high level of accuracy when taking into account NIHL-SG and NIHL-RG.

### Determining a Reliable Test Frequency for NIHL

Table 3 shows the mean accuracy and mean AUC obtained with the ML diagnostic models when various frequencies were used. Optimal performance was demonstrated by all 4 algorithms when 4 kHz combined with 12.5 kHz were used to detect NIHL; with these 2 frequencies, the maximum accuracy was 0.78 (adaptive boosting=0.79, multilayer perceptron=0.79, random forest=0.77, and support vector machine=0.78) and the AUC was 0.81 (adaptive boosting=0.83, multilayer perceptron=0.83, and random forest=0.7). When 4, 6 plus 12.5 kHz were used, the AUC was also high (0.81), but accuracy declined to 0.76. Therefore, we concluded that 4 and 12.5 kHz identified by the ML diagnostic model were the most reliable combination of frequencies to predict the NIHL.

**Table 3.** The mean accuracy and AUC^a^ of machine learning–based diagnostic models when various frequencies or frequency combinations were used.

Frequencies	AUC, mean (SD)	Accuracy, mean (SD)
Average 4 and 12.5 kHz	0.81 (0.02)	0.78 (0.02)
Average 4, 6, and 12.5 kHz	0.81 (0.02)	0.76 (0.01)
Average 4, 10, and 12.5 kHz	0.79 (0.02)	0.76 (0.02)
Average 4, 6, 10, and 12.5 kHz	0.80 (0.01)	0.75 (0.01)
Average 3, 4, 6, 10, and 12.5 kHz	0.80 (0.02)	0.74 (0.02)
Average 4 kHz	0.77 (0.03)	0.72 (0.03)
Average 12.5 kHz	0.77 (0.02)	0.76 (0.01)

a AUC: area under the curve.

### Clinical Characteristics of NIHL-SG and NIHL-RG Individuals

In some cases, the ML model placed individuals in the NIHL-SG or NIHL-RG contradicting the predicted PTA and their risk factors possibly due to genetic heterogeneity. Individuals who were correctly predicted by ML were used as a control group for further analysis of NIHL-SG and NIHL-RG. When using algorithms that do not involve probability values, such as Adaboost, the method of selecting extreme individuals with mean HTs can be used to identify the clinical characteristics of these individuals, thereby reducing potential bias caused by ML algorithms themselves. When the first 15% of individuals with the highest HTs from a susceptible portion of the NIHL-SG (n=79) were selected, the HTs at the susceptible frequencies exceeded the mean of the matched control group by more than 2 SDs. Using similar methods, the mean HTs in the NIHL-RG (n=139) were 2 SDs below the mean of the matched control group.

**Figure 2.**
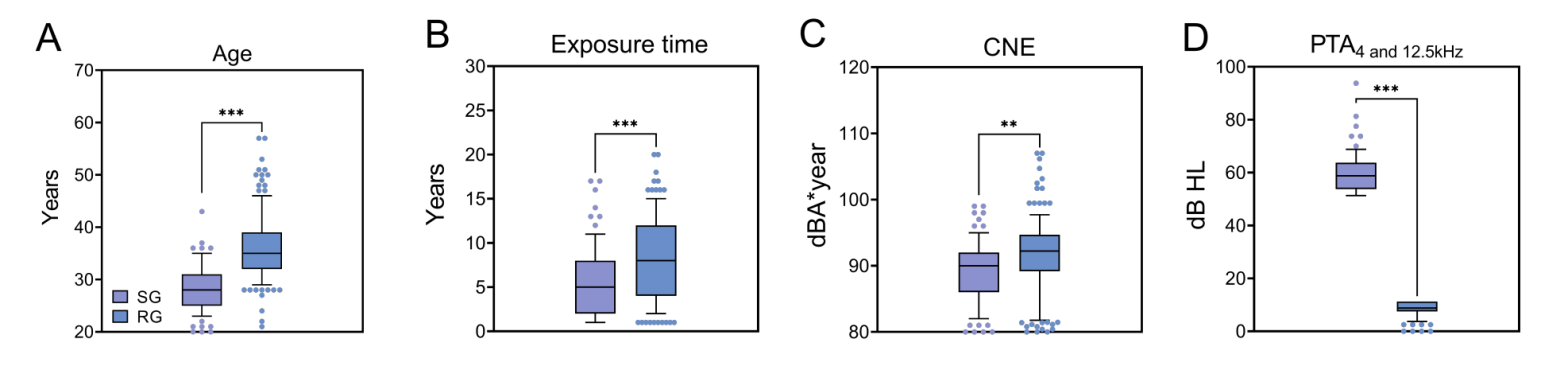
Demographic and audiometric characteristics of individuals in the noise-induced hearing loss (NIHL)–susceptible group (NIHL-SG) and NIHL-resistant group (NIHL-RG) classified by the optimized machine learning–based NIHL diagnostic model. Data show the differences between (A) NIHL-SG and NIHL-RG in age, (B) exposure time, (C) cumulative noise exposure (CNE), and (D) the mean pure tone audiometry (PTA) of binaural 4 and 12.5 kHz. Box plots show median and IQR and error bars represent the 10% and 90% percentiles. The *P* values are from the results of the Mann-Whitney test. *P<.05, **P<.01, and ***P<.001.

The characteristics of individuals in NIHL-SG and NIHL-RG are summarized in Table 4. HTs of the NIHL-SG were significantly higher than the RG at all frequencies (0.5‐2 kHz; *P*<.001; 3 kHz; *P*<.001; 4 kHz; *P*<.001; 6 kHz; *P*<.001; 10 kHz; *P*<.001; 12.5 kHz; *P*<.001; 4 and 12.5 kHz; *P*<.001). Workers in the NIHL-SG were younger than the NIHL-RG (Table 4, Figure 2A; *P*<.001). Workers in the NIHL-SG had a shorter noise exposure duration (Table 3, Figure 2B; *P*<.001) and a lower CNE level (Table 3, Figure 2C; *P*<.001).

**Figure 3.**
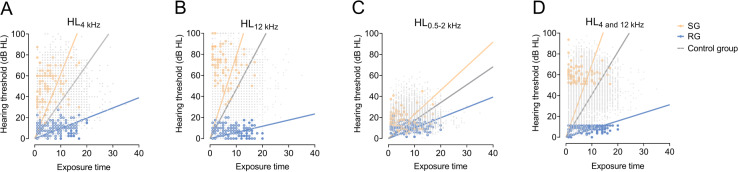
Scatterplots were constructed at frequencies of 4 kHz, 12.5 kHz, 0.5‐2 kHz and combined 4 and 12.5 kHz showing the hearing thresholds as a function of exposure time for each participant. The solid lines are fitting lines with an intercept of 0. HL: hearing level; RG: resistant group; SG: susceptible group.

At frequencies of 4, 12.5, 0.5‐2, and 4 and 12.5 kHz, the NIHL-SG showed more rapid growth of HL over time with slopes of 6.16, 7.82, 2.3, and 7 dB/year. The control group by contrast had shallower slopes of 3.52 dB/year at 4 kHz, 4.68 dB/year at 12 kHz, 1.71 dB/year at 0.5‐2 kHz, and 4.70 dB/year at 4 plus 12.5 kHz. The slopes of the NIHL-RG were even shallower with values of 0.98 dB/year at 4 kHz, 0.58 dB/year at 12 kHz, 0.99 dB/year at 0.5‐2 kHz, and 0.78 dB/year at 4 kHz plus 12.5 kHz (Figure 3). The slope of hearing loss per year was greatest for the NIHL-SG followed by the control group and the NIHL-RG (Figure 3).

**Table 4.** Demographic and audiometric characteristics of individuals in the NIHL^a^-susceptible group (NIHL-SG) and NIHL-resistant group (NIHL-RG) classified by the optimized machine learning–based NIHL diagnostic model.^b^

Characteristics	Susceptible group (n=79)	Resistance group (n=139)	*P* value
**Sex, n (%)**			<.001
Male, n (%)	68 (86.1)	126 (90.6)	
Female, n (%)	11 (13.9）	13 (9.4）	
Age (year), median (IQR)	28.0 (25.0‐31.0)	35.0 (32.0‐39.0)	<.001
Exposure time (year), median (IQR)	5.0 (2.0‐8.0)	8.0 (4.0‐12.0)	<.001
CNE^c^ (dBA-year), median (IQR)	90.0 (86.0‐92.0)	92.2 (89.2‐94.7)	<.001
**PTA**^**d**^ **(dB HL), median (IQR)**			
Average 0.5‐2 kHz	18.3 (14.2‐23.3)	10.8 (8.3‐14.2)	<.001
Average 3 kHz	35.0 (23.8‐50.0)	10.0 (7.5‐12.5)	<.001
Average 4 kHz	52.5 (37.5‐60.0)	10.0 (7.5‐12.5)	<.001
Average 6 kHz	52.5 (35.0‐55.0)	7.5 (5.0‐15.0)	<.001
Average 10 kHz	57.5 (42.5‐72.5)	7.5 (2.5‐10.0)	<.001
Average 12.5 kHz	70.0 (60.0‐80.0)	7.5 (2.5‐10.0)	<.001
Average 4 and 12.5 kHz	58.8 (53.8‐63.8)	8.8 (7.5‐11.3)	<.001

a NIHL: noise-induced hearing loss.

b The *P* values are from the results of the Mann-Whitney test. Statistical significance defined as *P*<.05.

c CNE: cumulative noise exposure.

d PTA: pure tone audiometry.

## Discussion

### Principal Findings

Among the methods for identifying individuals in the NIHL-SG and NIHL-RG, the ML-based NIHL diagnostic model with misclassified subjects was found to be the optimal method based on various characteristics believed to define these groups (ie, face validity). The accuracy of the ML diagnostic model for distinguishing the NIHL-SG from the NIHL-RG was highest when using 4 plus 12.5 kHz, suggesting that the mean HTs of these 2 frequencies could be an especially useful method to detect early onset hearing loss; however, this would require clinicians to routinely include testing at the EHFs rather than simply relying on the clinical audiogram.

Creating a standardized and effective method to identify susceptible and resistant individuals could help to advance research on NIHL (eg, identifying the genetics of NIHL susceptibility and resistance) and aid in its prevention. There is currently a lack of agreement among existing models regarding the most effective procedure to identify NIHL-susceptible or NIHL-resistant individuals. The literature indicates a linear increase in NIHL during the initial decade of exposure, followed by a nonlinear pattern thereafter [29]. Compared to linear and other regression models, the effectiveness of the ML for identifying individuals highly susceptible to developing NIHL may be related to their greater ability to detect nonlinear relationships between NIHL and multiple risk factors.

Our optimized ML model for detecting individuals either highly susceptible or highly resistant to NIHL could provide researchers with a powerful tool for studying the biological bases of NIHL, as well as for identifying lifestyle or environmental factors that enhance or suppress NIHL. Comparing the genetic makeup of individuals in the NIHL-SG and NIHL-RG to the general population could aid in the identification of genes or gene clusters that make some individuals resistant and others susceptible to NIHL [30,31]. Similarly, a more in-depth comparison of the physical characteristics, lifestyles, and medical histories of individuals in the NIHL-SG and NIHL-RG could reveal heretofore unknown variables that promote or impede the development of NIHL. Surveys of an individual’s lifestyle such as music listening habits, sleep patterns, use of noisy devices, and medication history could provide useful information and insights.

There is still some debate regarding the most susceptible frequency for identifying the early stage of NIHL. In the studies of risk evaluation for NIHL, the importance of several frequencies (including 3, 4, and 6) has been identified [32-34]. One biological reason for this is that the resonant frequency for the ear canal lies near 3‐4 kHz; sounds near this frequency are amplified roughly 10‐15 dB by the time these frequencies are transferred from the external environment to the tympanic membrane [35]. Another factor is that the upper frequency of clinical audiograms used to screen for NIHL is ≤ 8 kHz, this clinical procedure prevents researchers from exploring the growth of NIHL at the EHFs. The EHFs are most susceptible to age-related hearing loss [36,37]. However, there is growing recognition that the EHFs may also be highly susceptible to NIHL [38]. Others suggest that the proportion of individuals with notches at 3‐6 kHz is not overwhelming in noise-exposed individuals [39]. Some have argued that the EHFs are very effective at detecting early noise-induced hearing changes [40-42]. In the study of our group, the importance of EHFs (eg, 10 and 12.5 kHz) has also been confirmed [26]. However, because most audiometers have limited output at high frequencies, it is possible that frequencies above 12.5 kHz may be even more sensitive at detecting early NIHL [17]. In this study, various combinations of those frequencies were examined in the attempt to find 1 or 2 sensitive indicators, instead of using multiple indicators spreading to many frequencies. Interestingly, the ML diagnostic model attained peak discrimination and accuracy levels when using the average HTs at 4 and 12.5 kHz. The amalgamation of a traditional frequency with 12.5 kHz appears promising in detecting and discriminating individuals in the NIHL-SG and NIHL-RG.

The clinical characteristics of the individuals in the NIHL-SG and NIHL-RG provide a foundational standard for future research. In our ML-based NIHL diagnostic model, we selected the top 15% extreme values to minimize false positives. In the absence of a unified standard, our selection of the 15% extreme values approximates 2 SDs from the mean. It is not a fixed value but can be adjusted based on different data characteristics. It is important to note, however, that as the range of these extreme values increases, so does the risk of false positives.

### Limitations

There are some limitations to our study. The 15% in our extreme data samples predominantly consisted of males, a fact that largely reflects the predominance of males among all shipyard workers. This of course, likely introduced a gender bias in our findings, an issue that needs to be addressed in future studies. Additionally, although we calculated the growth rates of HL with exposure time using cross-sectional data, future efforts at assessing HL growth rates would benefit by evaluating longitudinal data beginning at an early age before the onset of hearing loss and prior to the entry of employees into noisy work environments. Because young individuals likely enter the workforce with a preexisting hearing loss at 12‐16 kHz and because commercial audiometers have a limited maximum output at these frequencies, it is difficult to measure the growth of NIHL across time in the workplace because of saturation effects. Although we excluded workers with a history of noise exposure, it is unclear whether some individuals who entered the study were unaware or unable to recall their history of exposure to recreational and environmental noise. Technological advances using cell phones and dedicated apps could conceivably make it possible to conduct yearly hearing assessments among a large cohort of individuals in order to track the growth of hearing loss with advancing age prior to the time when individuals enter the workforce.

### Conclusions

Using a large sample of auditory data from shipyard workers together with questionnaires and extensive demographic data, we compared various analytic methods for identifying individuals who were resistant or susceptible to shipyard noise. Our results suggest that our optimized ML diagnostic model with misclassified subjects, which assesses HTs at the EHFs in addition to the traditional audiometric frequencies, is the most effective method for identifying individuals highly susceptible or resistant to NIHL. Having identified individuals susceptible and resistant to NIHL with this method, future studies could search for genetic, environmental, and lifestyle factors that contribute significantly to the growth of NIHL.

## Supplementary material

10.2196/60373 Multimedia Appendix 1 Detailed content on audiological evaluation, noise exposure estimation, and an in-depth review of published methods.

## Abbreviations

AUC: area under the curve
CNE: cumulative noise exposure
EHF: extended high frequency
HT: hearing threshold
ML: machine learning
NIHL: noise-induced hearing loss
NIHL-RG: noise-induced hearing loss resistant group
NIHL-SG: noise-induced hearing loss susceptible group
