# Supplementary material for: Optimizing a Classification Model to Evaluate Individual Susceptibility in Noise-Induced Hearing Loss: Cross-Sectional Study
Source: JMIR Public Health Surveill. 2024 Nov 14;10:e60373. doi: 10.2196/60373 (PMC11615998; doi:10.2196/60373)

Methods

**Audiological Evaluation and Noise Exposure Estimation**

Hearing evaluation was performed by a qualified medical assistant. Otoscopy inspection was carried out to rule out blockage of the external ear. Tympanometry was tested with a TympStar tympanometer (Grason-Stadler; Eden Prairie, MN, USA) to ensure the normal middle ear impedance, which was indicated by type A tympanogram (peak between −100 and +100 daPa). Pure tone audiometry (PTA, 0.25-16 kHz ) was carried out by a qualified medical assistant in a soundproof chamber with background noise below 25 dB(A). At least 12 hours of rest after the last work shift were given when PTA was tested. Air-conducted pure-tone thresholds were tested with a Type 1066 manual audiometers (Natus Hearing & Balance; Taastrup, Denmark). TDH-39 headphones (Telephonics; Farmingdale, NY, USA) were used for the test in conventional frequency range (0.25-8kHz), and Sennheiser (Wedemark, Germany) HDA-300 headphones for the extended high frequencies (EHFs) (10-16 kHz). The audiometers underwent annual calibration in accordance with the ISO 389-5-2006 standard. All threshold measurements were documented in dB hearing level (HL). Participants who did not respond at the maximum output of the audiometer for EHFs (110, 100 and 65 dB for 10, 12.5 and 16 kHz respectively) were excluded from the analysis to eliminate saturation effects.

Industrial noise levels were assessed using an ASV5910-R digital recorder (Aihua Instruments; Hangzhou, China) across different work areas, adhering to the national standard of China [19]. The long-term equivalent (Leq) noise level was adopted as the primary exposure metric and measured three times at each spot. The mean Leq value in each spot was transformed into 8h of continuous equivalent A-weighted sound pressure level. Evaluation of noise exposure employed the CNE formula: CNE = Leq-8h + 10*log(T), where Leq-8h signifies the equivalent continuous sound level for 8 hours, and T denotes the exposure time [20].

**Review the published methods**

An initial literature search was conducted on PubMed and Web of Science, yielding 597 records related to NIHL. After a comprehensive examination of the details, six eligible studies were finally grouped into one of three models based on the approach.

**Model 1** incorporates three methods (1-3) that defined NIHL-SG and NIHL-RG based on extremes HTs within subgroups: **Method 1** identified the 20% most extreme HTs means in the left ear at 4 and 6 kHz within nine exposure time/noise intensity ranges (PTAmL4&6kHz20%, table 2) [9]. **Method 2** divided subjects into subgroups by age and noise intensity, then classified the 10% with the worst and best HTs in the left ear at 3 kHz as the NIHL-SG and NIHL-RG respectively (PTAL3kHz10%, table 2) [10]. **Method 3** similarly classified those with the 10% worst and best HTs in the left ear at 4 and 6 kHz as NIHL-SG and NIHL-RG (PTAL4&6kHz10%, table 2) [11].

**Model 2** included two linear modeling methods (4-5). **Method 4** used simple linear regression (LR) of bilateral HTs at 3, 4, and 6 kHz versus CNE, assigning those with the 10% highest residuals (measured minus predicted) and 10% lowest residuals to the NIHL-SG and NIHL-RG respectively (PTAm3,4&6kHz10%R, table 2)[13]. **Method 5** utilized a quadratic regression analysis; the NIHL-SG was defined as the 20% with the largest residuals, the 20% with the smallest residuals formed the NIHL-RG (PTAm3,4&6kHz20%Q, table 2)[12].

**Model 3** employed four ML algorithms (**Method 6**, table 2), using age, sex, CNE, smoking, and alcohol drinking status as input variables, to predict hearing outcomes[8]. The algorithms assigned susceptible status to individuals predicted to have better hearing, but actually exhibited worse hearing; those predicted to have worse hearing, but actually exhibited better hearing were assigned resistant status. The selection for the extreme individuals were restricted to the misclassified subjects by all algorithms. The NIHL-SG and NIHL-RG were comprised of 150 individuals with the largest probability values in each misclassified category provided by ML algorithms.

Results

**Validation of previous screening models**

Validation of Model 1 with extreme thresholds at 3, 4 and/or 6 kHz

In **Method 1**, 3276 individuals met the inclusion and exclusion criteria. The mean age of the NIHL-SG (n=655) was about 7 years older than the NIHL-RG (n=655; Figure S1a, table 2, 38.0 [32.0-44.0] versus 31.0 [27.0-36.8] years, *p*<0.001). The average noise-exposure duration was approximately 10 years in the NIHL-SG, significantly greater than that of the NIHL-RG (Figure S1b, table 2, 10.0 [7.0-14.0] versus, 7.0 [4.0-12.0] years, *p*<0.001). CNE values were significantly greater in the NIHL-SG than the NIHL-RG (Figure S1c, table 2, 93.4 [91.0-96.2] versus 91.4 [87.4-94.4] dBA-year, *p*<0.001), PTA values at 4 and 6 kHz were significantly greater in the NIHL-SG than the NIHL-RG (Figure S1d, table 2, 55.0 [47.5-62.5] versus 10.0 [7.5-12.5] dB, *p*<0.001).

For **Method 2** analysis, only 681 individuals were included. There was no significant difference in CNE between the NIHL-SG (n=68) and NIHL-RG (n=68; Figure S1g, table 2, 96.3 [94.0-104.5] versus 96.2 [93.2-98.2] dBA-year, *p*=0.064). The individuals classified as NIHL-SG on average were about 6 years older than those in NIHL-RG (Figure S1e, table 2, 48.0 [41.25-51.8] versus 42.0 [36.0-51.0] years, *p* =0.006). They also showed a slightly longer time of exposure (Figure S1f, table 2, 16.0 [15.0-20.0] versus 16.0 [15.00-18.0] years, *p*=0.032) but a greater PTA at 3 kHz (Figure S1h, table 2, 65.0 [60.0-70.0] versus 10.0 [5.0-10.0] dB, *p*<0.001).

For **Method 3**, a total of 5290 individuals were included in the analysis. The NIHL-SG (n=529) was significantly older (almost 20 years on average; Figure S1i, table 2, 47.0 [40.0-52.0] versus 31.0 [26.0-36.0] years, *p*<0.001), had significantly larger CNE (Figure S1k,, table 2, 95.9 [92.2-103.7] versus 91.0 [87.4-93.4] dBA-year, *p*<0.001), significantly longer duration of noise exposure (Figure S1j, table 2, 9.0 [6.0-12.0] versus, 5.0 [3.0-10.0] years, *p*<0.001) and notably poorer hearing than the NIHL-RG (n=529; Figure S1l, table 2, 70.0 [65.0-75.0] versus 7.5 [5.0-10.0] dB, *p*<0.001).

All individuals in the NIHL-SG selected by Model 1 were older, had longer exposure time and higher CNE than those in NIHL-SG which is inconsistent with known characteristics (i.e., poor validity). Several issues related to the analysis are of concern. There is no uniform criterion to define the age range or noise intensity range for different categories. Additionally, it unclear how many or what percent of individuals should be selected to form the NIHL-SG and NIHL-RG. The ability of Model 1 to identify individuals in the NIHL-SG and NIHL-RG is limited due to the potential influence of manual sorting.

Validation of Model 2- linear regression CNE versus mean HTs

The 5460 subjects with CNE levels above 80 dBA were included in the analysis of **Method 4**. The bilateral HTs at 3, 4, and 6 kHz versus CNE was significantly correlated (R^2^=0.102, *p*<0.001). Large differences were observed between the NIHL-SG (n=546) and NIHL-RG (n=546). Workers in the NIHL-RG were approximately 10 years younger than those in the NIHL-SG (Figure S2a, table 2, 35.0 [28.5-41.5] versus 46.0 [40.0-50.0] years, *p*<0.001). The NIHL-SG had slightly longer exposure times (Figure S2b, table 2, 8.0 [5.0-12.0] versus 7.0 [4.0-11.0] years, *p*=0.013), significantly lower noise exposure levels (Figure S2, table 2, 93.2 [90.2-98.0] versus 95.9 [92.5-100.7] dBA-year, *p*<0.001) but higher HTs at 3, 4, and 6 kHz (Figure S2d, table 2, 64.2 [58.3-70.0] versus 18.8 [11.3-27.5] dB, *p*<0.001) compared to the NIHL-RG.

For **Method 5**, 2315 individuals met the inclusion and exclusion criteria. Quadratic regression of bilateral HTs at 3, 4, and 6 kHz versus CNE was significantly correlated (R^2^=0.078, *p*<0.001). There were no exposure duration (Figure S2f, table 2, 9.0 [6.0-13.0] versus 9.0 [5.0-13.0] year, *p*=0.825) and no CNE (Figure S2g, table 2, 101.1 [96.6-103.7] versus 100.7 [97.7-104.2] dBA-year, *p*=0.18) differences between the NIHL-SG (n=463) and NIHL-RG(n=463). The NIHL-SG demonstrated a greater average hearing loss nearly 47 dB at 3, 4, and 6 kHz compared to the NIHL-RG (Figure S2h, table 2, 61.7 [55.8-67.5] versus,15.0 [11.7-18.3] dB, *p*<0.001); however, this may be related to the fact that the individuals in the NIHL-SG were significantly older than the NIHL-RG (FigureS2e, table 2, 49.0 [44.0-53.0] versus, 40.0 [33.0-48.1] years, *p*<0.001).

Although Model 2 showed that HTs were correlated with CNE, lower R^2^ values indicate that these models have not precisely identified individuals highly susceptible or resistant to NIHL.

Validation of Model 3 with ML

The ML model achieved an average accuracy of 0.74 and an average AUC of 0.80. Workers in the NIHL-SG (n=150) were younger than those in the NIHL-RG (n=150; Figure S3a, table 2, 32.0 [30.0-34.0] versus 48.0 [45.0-51.0] years, *p*<0.001), received lower noise exposure levels (Figure S3c, table 2, 91.5 [88.3-94.7] versus 96.2 [92.2-101.7] dBA-year, *p*<0.001), had shorter noise durations (Figure S3b, table 2, 6.0 [3.0-10.0] versus 9.0 [5.0-12.0] years, *p*<0.001) but greater HTs (Figure S3d, table 2, 33.8 [29.0-43.0] versus 21.0 [16.9-23.6] dB, *p*<0.001) than those in the NIHL-RG .

The misclassified subjects selected by the ML diagnostic model for the NIHL-SG and NIHL-RG were consistent with their expected clinical characteristics (i.e., strong face validity). The ML model also maintained a high level of accuracy when taking into account NIHL-SG and NIHL-RG.

**Figure S1.** Demographic and audiometric characteristics of subjects in the NIHL susceptible group (NIHL-SG) and NIHL resistant group (NIHL-RG) classified by Method 1 (a-d), Method 2 (e-h) and Method 3 (i-l). Box plots show median and IQR and error bars represent the 10% and 90% percentiles. Data show the differences between NIHL-SG and NIHL-RG in age (a, e, i), exposure time (b, f, j), cumulative noise exposure (CNE) (c, g, k), and the mean Pure tone audiometry (PTA) of 4 and 6 kHz (d, l) and 3 kHz (h). The significance symbols are as follows: * p < 0.05, ** p < 0.01, *** p < 0.001, ns: not significant.


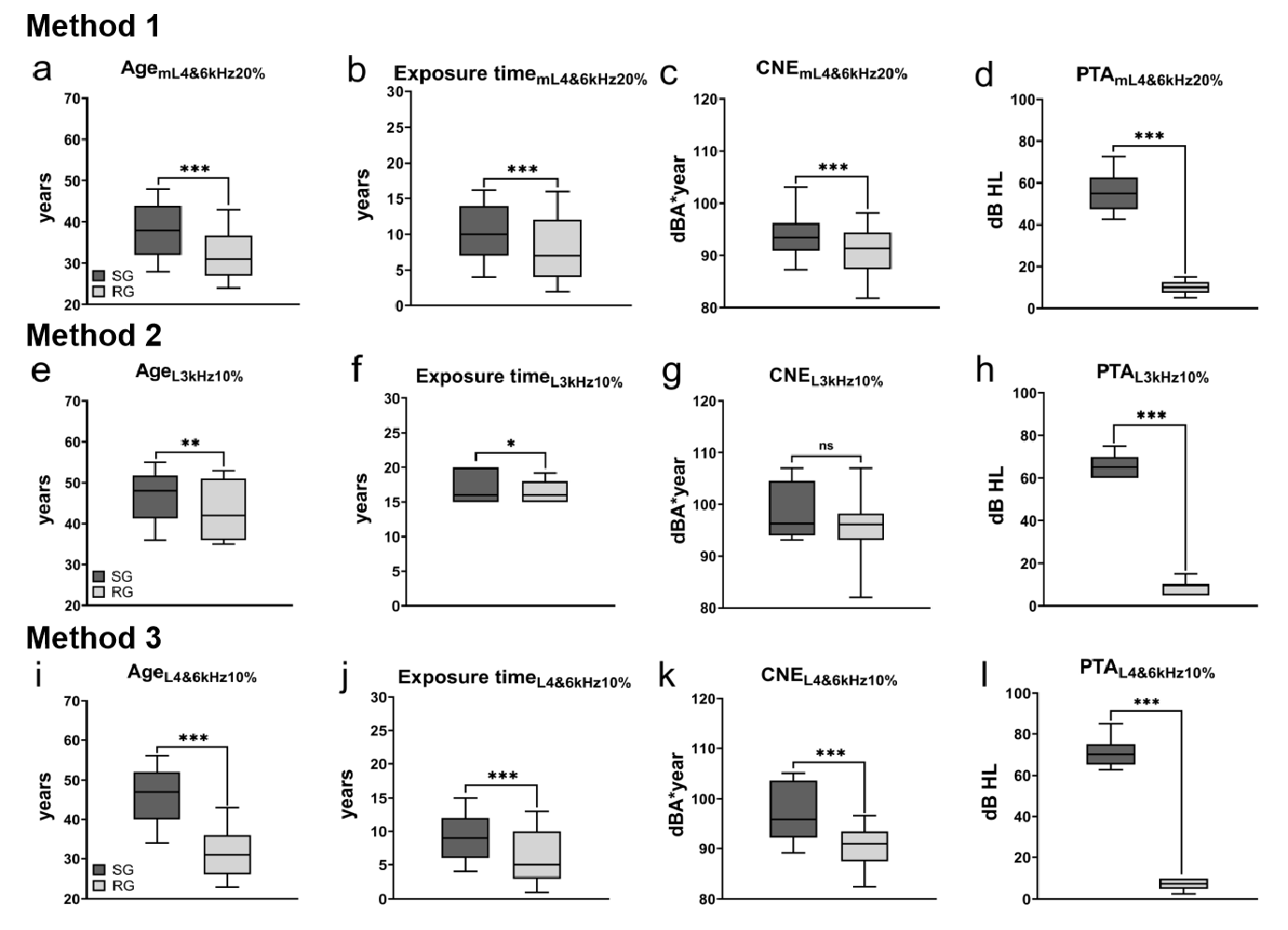


**Figure S2.** Demographic and audiometric characteristics of subjects in the NIHL susceptible group (NIHL-SG) and NIHL resistant group (NIHL-RG) classified by according to Method 4 (a-d) or Method 5 (e-h). Box plots show median and IQR and error bars represent the 10% and 90% percentiles. Data show the differences between NIHL-SG and NIHL-RG in age (a, e), exposure time (b, f), cumulative noise exposure (CNE) (c, g), and the mean Pure tone audiometry (PTA) of 3, 4, and 6 kHz (d, h). The significance symbols are as follows: * p < 0.05, *** p < 0.001, ns: not significant.


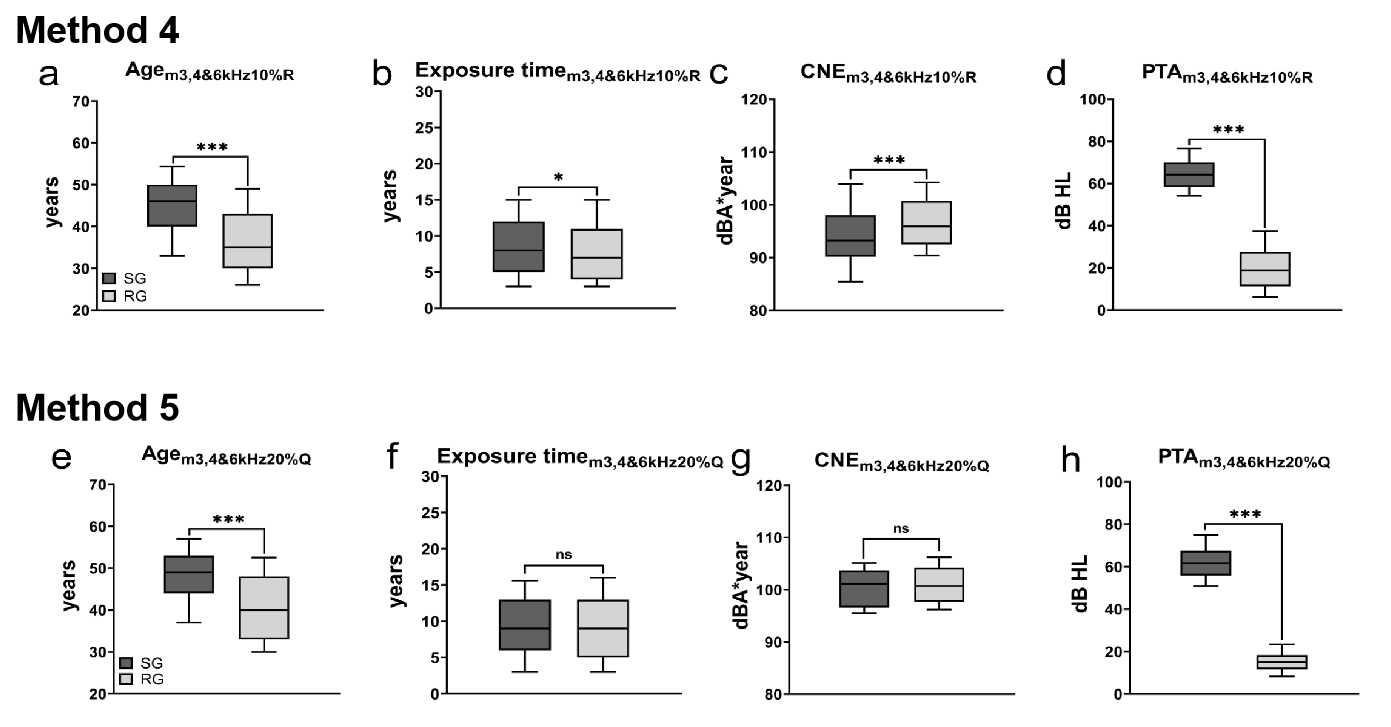


**Figure S3.** Demographic and audiometric characteristics of subjects in the NIHL susceptible group (NIHL-SG) and NIHL resistant group (NIHL-RG) classified by according to Method 6 (a-d). Box plots show median and IQR and error bars represent the 10% and 90% PTA of target frequency. Data show the differences between NIHL-SG and NIHL-RG in age (a), exposure time (b), cumulative noise exposure (CNE) (c), and the mean Pure tone audiometry (PTA) of 3, 4, and 6 kHz plus 10 and 12.5 kHz (d). *** p < 0.001.


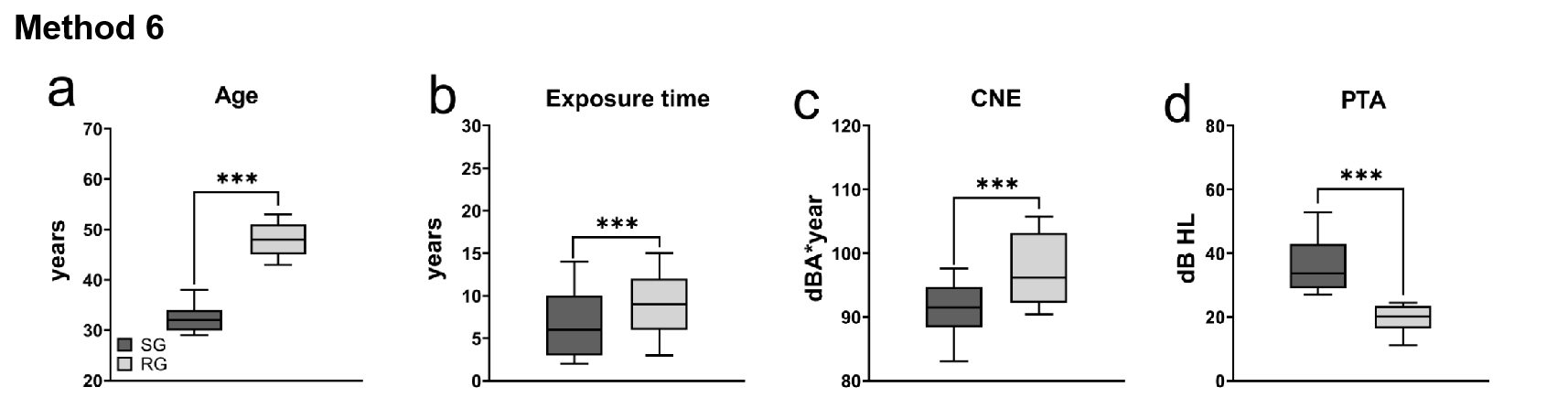

Supplement: Multimedia Appendix 1 [file publichealth-v10-e60373-s001.docx]
